# β-Sheet Structure within the Extracellular Domain of C99 Regulates Amyloidogenic Processing

**DOI:** 10.1038/s41598-017-17144-0

**Published:** 2017-12-07

**Authors:** Yi Hu, Pascal Kienlen-Campard, Tzu-Chun Tang, Florian Perrin, Rémi Opsomer, Marie Decock, Xiaoshu Pan, Jean-Noel Octave, Stefan N. Constantinescu, Steven O. Smith

**Affiliations:** 1 Department of Biochemistry and Cell Biology, Stony Brook University, Stony Brook, NY 11794 USA; 2 Institute of Neuroscience, Université catholique de Louvain, Brussels, 1200 Belgium; 3 Ludwig Institute for Cancer Research and de Duve Institute, Université catholique de Louvain, Brussels, 1200 Belgium

## Abstract

Familial mutations in C99 can increase the total level of the soluble Aβ peptides produced by proteolysis, as well as the Aβ42/Aβ40 ratio, both of which are linked to the progression of Alzheimer’s disease. We show that the extracellular sequence of C99 forms β-sheet structure upon interaction with membrane bilayers. Mutations that disrupt this structure result in a significant increase in Aβ production and, in specific cases, result in an increase in the amount of Aβ42 relative to Aβ40. Fourier transform infrared and solid-state NMR spectroscopic studies reveal a central β-hairpin within the extracellular sequence comprising Y10-E11-V12 and L17-V18-F19 connected by a loop involving H13-H14-Q15. These results suggest how familial mutations in the extracellular sequence influence C99 processing and provide a structural basis for the development of small molecule modulators that would reduce Aβ production.

## Introduction

**Figure 1:**
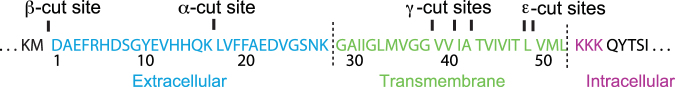
Sequence and processing of C99. (**A**) Sequence of the extracellular (blue) and transmembrane (green) domains of C99. C99 or the β-CTF corresponds to residues 1–99. The C-terminal three residues of C55 (i.e. the first 55 residues of C99) are shown in purple. The α-, β-, γ- and ε-cleavage sites are shown.

The amyloid-β precursor protein (APP) is a type I membrane protein with a bulky extracellular domain, a single transmembrane (TM) helix and a relatively short, disordered C terminal tail. It is widely expressed in different cell types, including neurons^1^. APP is processed by the successive cleavage of two proteases. The first cleavage is carried out between its extracellular and TM domains by either α- or β-secretase, generating N-terminal soluble fragments (soluble αAPP or soluble βAPP, respectively) and membrane-anchored C-terminal fragments (α- or β-CTF, respectively). The γ-secretase complex further cleaves the TM domain of the α- and β-CTFs (Fig. 1). Cleavage of the β-CTF (also called C99) generates the amyloid-β (Aβ) peptides with various lengths, with Aβ40 peptide being the most dominant. Among the Aβ peptides, the 42-residue Aβ42 peptide is considered to be the most toxic and prone to aggregation^2,3^, and is the principal component of amyloid plaques in AD patients^4^.

It is well known that mutations in the extracellular sequence influence cleavage by the β-secretases. There is a cluster of amino acids near the β-secretase cleavage site at Lys595-Ala598 where mutations increase β-secretase cleavage^5,6^. Engineered mutations within this cluster are widely used to enhance the production of Aβ from APP^7^. (In the following, we use the β-CTF numbering, which also coincides with the numbering of the Aβ peptides. Asp1 is the first residue of the β-CTF or Aβ rather than Asp597, which corresponds to substrate numbering based on the APP695 isoform of APP.)

It is perhaps more surprising that mutations in the extracellular sequence also influence the cleavage of the β-CTF within its transmembrane domain by γ-secretase. The extracellular sequence is considerably distant from the γ-secretase cleavage site and is not conserved among different γ-secretase substrates. For example, the A21G (Flemish), E22Q (Dutch), E22G (Arctic), E22K (Italian), and D23N (Iowa) mutations^8–11^ in the extracellular sequence have very different effects on APP processing^12^ and Aβ peptide degradation^13^. While mutations at Ala21 increase Aβ secretion, mutations at Glu22 and Asp23 generally decrease the level of total secreted Aβ peptides^12,14,15^.

Immediately on the N-terminal side of Ala21 is the L17-V18-F19-F20 sequence. This sequence was previously shown to be part of an inhibitory motif that reduces the production of soluble Aβ peptides upon γ-secretase processing^14^. The level of soluble Aβ increases dramatically upon deletion or mutation of this motif. This increase was observed without a change in ε-cleavage, which produces the free APP intracellular domain (AICD)^14^. Peptides derived from this motif (e.g. Aβ17-21) are able to noncompetitively inhibit cleavage of TM peptides derived from APP suggesting that there is a binding site within the γ-secretase complex distinct from the active site that influences γ-secretase processing.

In contrast to mutation of Ala21 and the LVFF sequence, mutations in the G25-S26-N27-K28 region, which is a few residues downstream of Ala21, reduce the level of secreted Aβ40 and Aβ42. Specifically, mutations at Ser26 and Lys28 lead to a decrease in Aβ40 and Aβ42, without a corresponding loss of the AICD cleavage product, indicating that the mutations do not reduce overall γ-secretase cleavage^16^. Kukar *et al*.^17^ found that the K28A/K28Q mutations shift the major cleavage site to the position of Gly33 from Val40. These results suggest that the transition region between the extracellular and TM domains can have a substantial effect on the position of γ-cleavage within the TM domain.

In order to understand how extracellular mutations influence enzymatic cleavage within the TM domain of the β-CTF and/or the release of soluble Aβ, we use Fourier transform infrared (FTIR) and nuclear magnetic resonance (NMR) spectroscopy to establish the structure of the extracellular domain of the β-CTF and combine these results with structural and functional studies of extracellular mutants to assess how the substrate structure influences soluble Aβ production. Recently, we were able to show that the inhibitory LVFF sequence of the β-CTF has β-strand secondary structure^18^. Here we demonstrate that the LVFF and upstream YEV sequences associate to form a β-hairpin. We also identify a third β-component at the N-terminus that stabilizes the β-structure of the extracellular domain. A loss of β-structure within the extracellular domain correlates with an increase in secreted Aβ peptides. We then narrow down the elements within the LVFF motif that when mutated are responsible for the β-strand structure and the increase in Aβ secretion.

The increase in soluble Aβ that is observed upon mutation or deletion of the LVFF sequence can result either from an increase in processing of the β-CTF and/or a decrease in binding of the Aβ peptide to the plasma membrane. Previous studies have shown that the LVFF region of the β-CTF can bind to membranes^19^ suggesting that this sequence also mediates membrane binding of the cleaved Aβ peptides. To address the location of the LVFF sequence relative to the membrane bilayer, we target Phe19 and Phe20 within this sequence using ^31^P, ^13^C rotational-echo double-resonance (REDOR) NMR and fluorescence spectroscopy. REDOR NMR measurements can determine the proximity of ring- ^13^C-labeled Phe19 and Phe20 side chains to the ^31^P phosphate of the phospholipid head-groups, while fluorescence measurements on peptides containing either a F19W or F20W mutation can probe membrane binding on the basis of intrinsic tryptophan fluorescence.

Together these studies provide insights into how the extracellular sequence may influence the processing and release of soluble Aβ. The structure and folding of the N-terminal sequence are likely to be relevant not only for the release of the Aβ peptides from the γ-secretase complex, but also for the formation of soluble and membrane-bound Aβ oligomers, both of which are reported to be toxic to neuronal cells.

## Results

### Role of the Extracellular Sequence in C99 Processing

**Figure 2:**
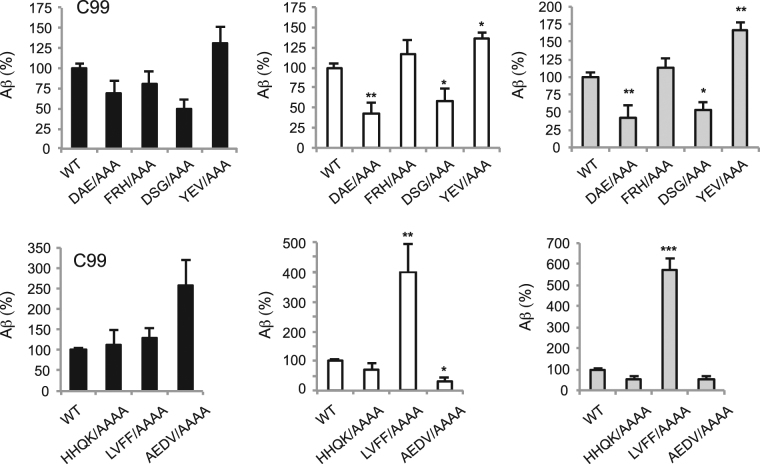
Alanine scanning mutagenesis of the extracellular domain of C99 reveals sequence specific increases and decreases in Aβ production. Comparison of the levels of Aβ38, Aβ40 and Aβ42 produced by γ-secretase cleavage of wild- type β-CTF and the corresponding N terminal alanine mutants. Values are the means ± S.E., n > 5; *p < 0.05; **p < 0.01; ***p < 0.001, compared with control.

To address the influence of the extracellular sequence on the processing of the β-CTF (referred to below as C99) to Aβ38, Aβ40 and Aβ42, we mutated the N-terminal residues from Asp1 to Val24 to alanine in groups of 3 or 4 amino acids (Fig. 2). Within the first 12 residues, alanine mutations of the D1-A2-E3 and D7-S8-G9 sequences lead to a reduction in the levels of secreted Aβ38, Aβ40 and Aβ42. In contrast, mutation of the Y10-E11-V12 sequence leads to increases in the secreted Aβ peptides, with the most significant increase in Aβ42. These results indicate that the structure or interactions of the most N-terminal residues of C99 influence γ-secretase processing.

Within the second 12 residues of the C99 sequence, mutation of the H13-H14-Q15-K16 and E22-D23-V24 sequences to alanine results in a slight decrease in the level of  soluble Aβ40 and Aβ42 peptides. However, mutation of the intervening L17-V18-F19-F20 sequence results in a significant increase in secreted Aβ40 and Aβ42. Comparison of the mutational results from Asp1 to Val24 clearly shows that the largest influence of mutation occurs at the LVFF sequence, but with unexpected alternation of regions that promote and inhibit amyloidogenic processing.

The increase in the secreted Aβ40 and Aβ42 upon mutation of the extracellular LVFF sequence is due in part to an increase in ε-cleavage at the C-terminus of the TM domain of the β-CTF, which releases the AICD. Measurements of AICD release using a luciferase reporter assay show a significant increase in ε-cleavage in the LVFF-to-AAAA mutant relative to the wild-type sequence and a corresponding increase in AICD^18^. These results indicate the influence of the LVFF-to-AAAA mutation is largely on γ-secretase processing rather than on α- or β-secretase processing^18^.

### Global Secondary Structure of C55 by FTIR Spectroscopy

**Figure 3:**
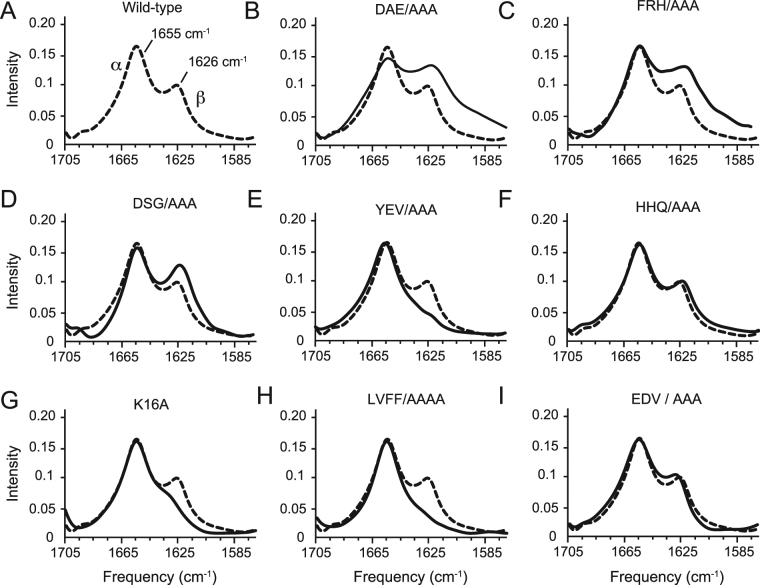
FTIR spectroscopy of wild-type and alanine mutants of C55. Comparison of FTIR spectra of wild-type C55 (**A**) reconstituted into membrane bilayers with spectra of the C55 protein containing alanine mutations at (**B**) DAE, (**C**) FRH, (**D**) DSG, (**E**) YEV, (**F**) HHQ, (**G**) K, (**H**) LVFF, and (**I**) EDV. The bilayers were formed with dimyristoylphosphocholine (DMPC) and dimyristoylphosphoglycerol (DMPG) in a molar ratio of 10:3. The molar ratio of C55:total lipid was 1:50.

The C55 peptide corresponds to the first 55 residues of the β-CTF or C99, and encompasses the extracellular sequence, the TM domain and an intracellular cluster of positive charges. This is the minimal sequence containing the entire extracellular and TM domains of C99 needed for γ-secretase processing^18^. The amide I vibration (1600–1700 cm^−1^) in FTIR spectra is a sensitive marker of protein secondary structure. The FTIR spectrum of C55 reconstituted into membrane bilayers (Fig. 3A) exhibits both an intense band at 1655 cm^−1^ corresponding to α-helical structure and a weaker band at 1626 cm^−1^ corresponding to β-strand or β-sheet structure. The α-helical band represents the membrane-spanning portion of C55 stretching from roughly Lys28 to Leu52 (Fig. 1)^18^, while the β-strand or sheet component is associated with the extracellular sequence of C55. We have previously shown that mutation of the LVFF motif resulted in loss of the β-strand component within the extracellular domains of both C55 and C99^18^. The β-strand structure was shown to be consistent with NMR chemical shifts within the LVFF sequence and shown to convert to α-helix in the presence of detergent (Fig. S1). We now probe whether the β-strand structure of the extracellular domain extends to the N-terminus, whether the β-sheet formed is intra- or intermolecular and whether disruption of this sequence correlates with changes in γ-secretase processing.

Figure 3 presents the FTIR spectra of C55 containing alanine mutations stretching from the N-terminal D1-A2-E3 sequence to the E22-D23-V24 sequence just before the TM domain. We observe a loss of the IR band at 1626 cm^−1^ in the LVFF to AAAA mutant (Fig. 3H) as observed previously^18^. However, we also observe loss of the 1626 cm^−1^ band upon mutation of YEV to alanine (Fig. 3E), but not upon mutation of the intervening HHQ sequence (Fig. 3F). We attribute this pattern to the presence of a β-hairpin in which the HHQ sequence forms a loop connecting antiparallel YEV and LVFF β-strands. At the N-terminus, mutation of DAE and FRH appears to broaden the 1626 cm^−1^ band (Fig. 3B and C). IR spectra of parallel samples of the DAE and FRH to AAA mutants (data not shown) reveal variability in the frequency and breadth of the 1626 cm^−1^ band, suggesting that the most N-terminal amino acids interact with the β-hairpin formed by the LVFF and YEV sequences to form a three-stranded β-sheet.

The FTIR measurements showing loss of β-sheet secondary structure in the YEV and LVFF mutants correlates with the processing results in Fig. 2 where mutation of the YEV and LVFF sequences leads to the largest increases in secreted Aβ.

### Phe19 and Phe20 are Critical Determinants of the β-Sheet Structure in C99

**Figure 4:**
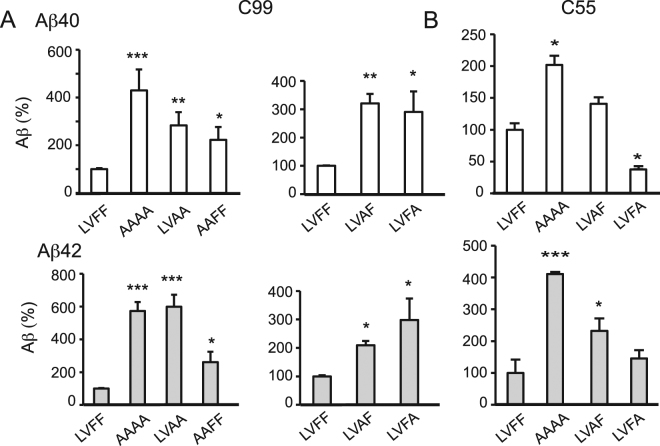
Influence of double and single mutants within the LVFF sequence on γ-secretase processing of C99 (**A**) and C55 (**B**). Values are the means ± S.E., n > 5; *p < 0.05; **p < 0.01; ***p < 0.001, compared with control.

To better define the residues that control the structure of the extracellular domain, single and double amino acid replacements were performed in the LVFF region of C99 (Fig. 4). Comparison of γ-secretase processing of the wild-type C99 sequence (LVFF) with that of the LVAA and AAFF mutants shows that the F19-F20 dipeptide has a slightly greater contribution to increased production of Aβ40 and Aβ42 than the L17-V18 dipeptide (Fig. 4A). For the F19-F20 dipeptide, the individual mutation of FA or AF results in comparable increases in Aβ40 and Aβ42.

Parallel studies were also undertaken on C55, the substrate used for the structural studies (Fig. 4B). For C55, mutation of LVFF to AAAA results in significant increase in secreted Aβ40 and Aβ42. However, with the individual amino acid changes, the F19A mutant leads to more secreted Aβ40 and Aβ42 than the F20A mutant.

**Figure 5:**
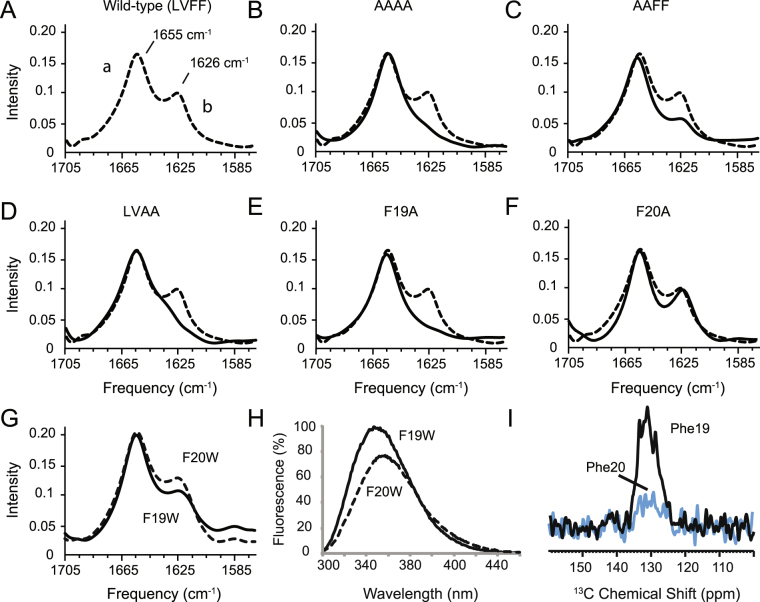
Contribution of Phe19 and Phe20 to β-sheet structure and membrane binding using single alanine mutants of C55. Comparison of FTIR spectra of wild-type C55 (**A**) reconstituted into DMPC:DMPG bilayers with spectra of the C55 protein containing mutations at (**B**) LVFF, (**C**) LV, (**D**) FF, (**E**) Phe19 and (**F**) Phe20. The molar ratio of the DMPC:DMPG was 10:3, and the molar ratio of C55:total lipid was 1:50. (**G**) Membrane penetration of Phe19 and Phe20 shows differences in the orientation of the LVFF phenylalanines. Comparison of FTIR spectra of wild-type C55 with C55 containing either the F19W (black) and F20W (dashed line) mutations shows that the mutations do not influence the C55 secondary structure. (**H**) Tryptophan fluorescence of F19W (black) and F20W (dashed line) C55. The fluorescence spectra were obtained with a 200:1 lipid to protein molar ratio. Experiments with 300:1 and 100:1 gave similar results. (**I**) ^13^C-^31^P REDOR NMR NMR measurements reveal membrane insertion of Phe19 within the LVFF sequence. ^13^C-observe, ^31^P-dephase REDOR spectra were obtained of wild-type C55 containing ^13^C-ring labeled Phe19 (solid-line) or ^13^C-ring labeled Phe20 (blue) reconstituted into DMPC:DMPG (10:3 molar ratio) bilayers.

FTIR measurements on the single and double mutants within the LVFF motif in C55 were also undertaken (Fig. 5). In comparison with wild-type C55 (LVFF), mutation of FF-to-AA (i.e. LVAA) results in a dramatic decrease in the 1626 cm^−1^ amide I vibrational band, indicating loss of β structure (Fig. 5D). In contrast, the LV-to-AA mutant (i.e. AAFF) behaves more like the wild-type LVFF sequence (Fig. 5C). We next undertook single mutations of the F19-F20 motif and found that the single F19A mutation results in complete loss of β structure (Fig. 5E). The single F20A mutation has a much milder phenotype (Fig. 5F). These data suggest that F19 is critical to the formation of β structure in the extracellular domain of C55.

### Phe19 Penetrates into the Membrane  Interface

Structural studies undertaken on C99 have shown that the highly hydrophobic LVFF sequence is buried in the hydrophobic region of membranes or micelles^19^. The membrane environment can have a strong influence on the folding of hydrophobic or amphipathic sequences. To address the location of the LVFF sequence relative to the membrane bilayer, we undertook both fluorescence and NMR studies. For the fluorescence measurements, we individually replaced Phe19 and Phe20 with tryptophan, whose fluorescence spectrum is sensitive to its chemical environment.

First, to confirm that the phenylalanine to tryptophan substitution does not disrupt the β-sheet structure in the region of Leu17-A21, FTIR studies were performed on the F19W and F20W mutants reconstituted into the DMPC/DMPG bilayers (Fig. 5G). Both FTIR spectra exhibit the 1626 cm^−1^ marker of β-sheet secondary structure. In contrast, comparison of the fluorescence spectra of the F19W and F20W mutants shows a blue shift and increase in the fluorescence curve from 357 to 346 nm (Fig. 5H) only for F19W. These changes indicate that the environment surrounding Trp19 of C55 is more hydrophobic than for Trp20 and suggest that Phe19 inserts into the head group region of the membrane bilayer, while Phe20 is more exposed to the aqueous hydrophilic environment outside of the bilayer.

NMR spectroscopy provides a second approach for comparing the ability of Phe19 and Phe20 to penetrate into the lipid headgroup region of the membrane bilayer. In the NMR experiments, we specifically label the Phe19 and Phe20 rings with ^13^C and measure their proximity to the ^31^P phosphate groups of the phospholipids via ^13^C-to-^31^P dipolar couplings using rotational echo double resonance (REDOR) NMR difference spectroscopy. The REDOR difference spectrum exhibits intensity changes when the ^13^C resonances of specifically ^13^C-labeled residues are within ~ 6–7 Å of a ^31^P phosphate^20^. Figure 5I compares the REDOR difference spectrum of C55 labeled with ring-^13^C-Phe19 with the REDOR difference spectrum of C55 labeled with ring-^13^C-Phe20. The strong intensity observed in the Phe19 ring-^13^C resonances in Fig. 5I is consistent with insertion of the Phe19 ring into the lipid head group region of the bilayer.

It is well known that the Aβ peptides bind to membrane bilayers^21^ and it is generally assumed that the binding is mediated by the hydrophobic C-terminal sequence. The observation that extracellular sequence of C55 binds to membrane bilayers raises the possibility that this sequence contributes to membrane binding in the context of the Aβ peptides. If this is correct, then the LVFF-to-AAAA mutation may reduce membrane binding and lead to an increase in soluble Aβ as in Figs 2 and 3. FTIR experiments on Aβ40 and Aβ42 show that the Aβ peptide rapidly adopts stable anti-parallel β-structure when added to a solution of large (100 nm diameter) unilamellar vesicles composed of DMPC (Fig. S2). Mutation of LVFF to AAAA results in Aβ40 and Aβ42 peptides with largely random coil structure, and decreases the ability of these peptides to bind to DMPC membrane bilayers (Fig. S3).

### Extracellular β-Sheet of C55 is Stabilized by Intramolecular Interactions

The structural and functional data described above suggest that the Y10-E11-V12 and L17-V18-F19 sequences form anti-parallel β-strands that associate to yield the observed β-hairpin structure. The antiparallel geometry is consistent with the weak 1695 cm^−1^ band in the wild-type spectrum, as well as isotope-induced shifts upon ^13^C labeling^18^. The H13-H14-Q15 to AAA mutation does not influence the wild-type structure, suggesting that it acts as a linker between the anti-parallel β strands. The C-terminal β-strand may start as early as Lys16, as the K16A mutation does disrupt the β structure (Fig. 3G). Since the side-chain orientation alternates by 180° in β-sheet structure, this suggests that Lys16, Val18 and Phe20 would lie on one side of the β-sheet, while Leu17 and Phe19 would lie on the other (and inserted into the headgroup region of the membrane). This further suggests that Tyr10 and Val12 are also inserted into the headgroup region of the bilayer while Glu11 is oriented away. The C55 D1-A2-E3 and F4-R5-H6 to AAA mutations both appear to generate more heterogeneity in the β structure as evidenced by the broadened amide I vibrations at 1626 cm^−1^ (Fig. 2B,C). In contrast, the D7-S8-G9 to alanine mutant peptide has an FTIR spectrum resembling wild-type (Fig. 2D), arguing that it has a role in bridging the N-terminus and the downstream β components. The mutations of D1-A2-E3 and F4-R5-H6 to alanine have the effect of increasing the overall hydrophobicity of these segments. One possibility is that these now newly generated hydrophobic segments interact with the hydrophobic LVFF sequence and further stabilize its structure. This would lead to the decrease in the amount of soluble Aβ observed in Fig. 2. These data suggest that the N-terminus only partially stabilizes the β-sheet structure, and that changes in this region either by mutation or by other interacting molecules might be able to prevent amyloidogenic processing.

**Figure 6:**
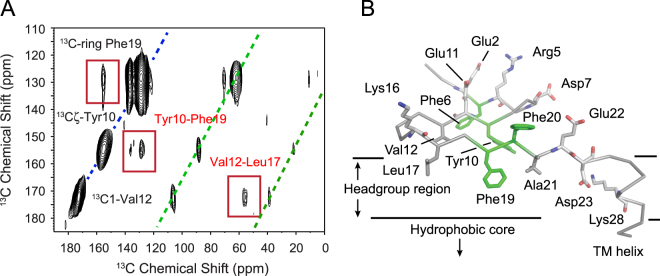
Structure of the extracellular sequence of C55. (**A**) Solid-state DARR NMR of intra-residue contacts between Val12 - Leu17 and Tyr10 - Phe19. The C55 peptide labeled with ^13^Cζ-Tyr10, ^13^C1-Val12, ^13^Cα-Leu17 and ^13^C-ring-Phe19 was reconstituted into DMPC:DMPG bilayers as previously described^18^. (**B**) Model of the C55 extracellular region based on FTIR and NMR data showing the regions of the β-secondary structure, Val12 - Leu17 and Tyr10 - Phe19 contacts, and orientations of Phe19-Phe20 relative to the membrane bilayer surface.

A model based on the FTIR and REDOR NMR data with the short three-residue LVF and YEV sequences as interacting β-strands with Phe19 oriented toward the lipid membrane is shown in Fig. 6. To test this model, two dimensional (2D) ^13^C dipolar assisted rotational resonance (DARR) NMR experiments were carried out on C55 labeled with ^13^Cζ-Tyr10, ^13^C1-Val12, ^13^Cα -Leu17 and ^13^C-ring-Phe19. As in the REDOR experiment, ^13^C…^13^C distances are obtained through measurements of dipolar couplings. The resonances along the diagonal of the 2D plot correspond to the one-dimensional (1D) NMR spectrum. Off-diagonal cross peaks arise from ^13^C sites that are separated in space by less than ~6 Å. A portion of the 2D DARR NMR spectrum of membrane-bound C55 is shown in Fig. 6A. Cross peaks are observed between ^13^C1-Val12 and ^13^Cα-Leu17, as well as between ^13^Cζ-Tyr10 and ^13^C-ring Phe19 consistent with the intra-molecular β-sheet formation within the extracellular N-terminus of C55.

The possibility arises that the KLVFFAE sequence on different peptides can pair together to form anti-parallel β-strands^22^. To verify that the LVFF and YEV sequences form an intra- rather than inter-molecular β-sheet structure, we introduced the E22K mutation into C55. If the inter-molecular β-sheet structure is correct, disrupting the salt bridge between Glu22 and Lys16 from the opposing strands might potentially dissociate the β-sheet structure. The FTIR spectrum of E22K shows that the β-sheet structure is not only conserved upon E22K mutation, but the intensity of the β-sheet signature band at around 1626 cm^−1^ is enhanced, suggesting a more stable anti-parallel β-sheet (Fig. S4). The increase in β-sheet structure in the E22K mutant is consistent with mutational studies at Glu22 and Asp23 that generally reveal a decrease in total secreted Aβ peptides upon mutation^12,14,15^.

FTIR spectra were also obtained of C39, corresponding to the extracellular and TM domains of the α-CTF. These data show that the β-strand IR band is lost upon deletion of the N-terminal 16 amino acids of C55 (Fig. S4) arguing that the LVFFAE sequence alone (i.e. in the context of the α-CTF) is not able to form anti-parallel structure.

## Discussion

The mechanism of intra-membrane cleavage of APP remains a mystery despite recent progress in establishing the structure of the γ-secretase complex. Here, we address the structure of the membrane-embedded γ-secretase substrate that generates the Aβ peptides associated with AD. We find that the extracellular sequence of C55, the minimal substrate processed by the γ-secretase complex to Aβ, is not disordered, but folds into a defined structure in the absence of γ-secretase. The LVFF and YEV sequences in the N-terminus of C55 contribute to a β-hairpin, in which the hydrophobic Leu17, Phe19, Tyr10 and Val12 residues are inserted into the head group region of the bilayer. This structure is stabilized by the N-terminus and constitutes a default brake to the production of soluble Aβ. Together, these results provide insights into γ-secretase processing of wild-type and mutant β-CTF into Aβ.

The mechanism by which the extracellular β-sheet structure impairs the release of the soluble Aβ peptide is not known. One possibility is that the β-sheet modulates interactions with the γ-secretase complex. For example, Tian *et al*.^14^ showed that deletion of the LVFFA sequence (L17-A21) increases the production of soluble Aβ at least 10-fold without a change in AICD release. The authors argued that there is an LVFF binding site on γ-secretase and that substrate binding inhibits γ-secretase activity. β-sheet may be required for this inhibitory interaction. Alternatively, the inhibitory interaction of the LVFF sequence may be with the membrane bilayer as suggested by the structural and mutational studies reported here. Of interest, it has recently been reported that pathologic amyloidogenic processing in neurons occurs mainly in late endosomes and lysosomes^23^. The acid pH present in the lumen of those organelles may disrupt the extracellular β-sheet of C99 due to protonation of residues that stabilize the β-sheet. Straub and coworkers^24^ have suggested the presence of a protonation switch involving Glu22 and Asp23, which may be neutralized at low pH and favor cholesterol binding. In our model, this would disrupt charge pairing with (for e.g.) Asp23 and Lys28. Comparison of FTIR spectra of C55 reconstituted into POPC:PS bilayers at pH 5 and pH 7 exhibit broadening and loss of intensity of the 1626 cm^−1^ β-sheet resonance consistent with disruption at lower pH (Fig. S5).

The structure of the extracellular sequence may influence its interaction with nicastrin. Studies several years ago found that the interaction of the N-terminal NH_3_ + of C99 with nicastrin enhances γ-secretase activity and argued that Glu333 on the nicastrin ectodomain was involved in substrate recognition^25^. Glu333 is 63 Å from the catalytic Asp385 of presenilin in the cryo-EM structure of the γ-secretase complex^26^. Such an interaction would require unraveling of the extracellular sequence. N-terminal mutations that disrupt the membrane bound β-sheet may facilitate unraveling of the N-terminus and possibly improve the ability of nicastrin to bind to the substrate and recruit it to the complex. We observe an increase in AICD release in the LVFF-to-AAAA mutant of C99 consistent with this as a possible mechanism. However, more recent studies on the Notch substrate do not reveal an interaction between nicastrin and the extracellular sequence^27^. Rather, substrate binding to the γ-secretase complex was found to be driven by interactions with the TM domain.

Finally, the structure and membrane interactions of the extracellular domain of C99 may influence dimerization and/or the orientation of the TM helix, and correspondingly influence its ability to be cleaved by the γ-secretase complex. Familial mutations in the TM domain have previously been suggested to influence dimerization and result in an increase in the Aβ42/40 ratio^28^, although recent cross-linking studies indicate that dimerization blocks γ-secretase activity^29^. In our systems, the C55 peptides exist in a monomer-dimer equilibrium^18^. The dimer structure is mediated by the G^29^xxxG^33^xxxG^37^ interface within the TM domain (Fig. S6A), and we have recently shown that the familial A21G mutation leads to an increase in dimerization of C55 and an increase in total secreted Aβ peptides^18^. Importantly, this mutation destabilizes the LVFF β-sheet structure.

The dimer interface mediated by GxxxG motifs in C55 differs from the interface mediated by the G^38^xxxA^42^ motif first suggested using computational methods^28^ and later observed using solution NMR spectroscopy on TM peptides beginning with Gln15^30^. In this regard, removal of first 16 amino acids of C55 to form the C39 peptide, which serves as our model of the α-CTF, disrupts the extracellular β-sheet structure when reconstituted into membrane bilayers (Fig. S4A) and results in an increase in TM dimerization (Fig. S6B).

On the basis of our studies, small molecules or peptides that disrupt the membrane bound β-sheet structure in the β-CTF would be expected to increase Aβ production, whereas therapeutics that stabilize β-sheet structure would reduce Aβ production. Nicolau and coworkers found that palmitoylation of Aβ1-15 targeted this sequence to liposomal membranes and induced β-structure^31,32^. Antibodies formed against this membrane-bound sequence restored memory defects in mice^33^. Together these studies support the idea that membrane binding may induce β-sheet structure in the N-terminus of the β-CTF and that this β-sheet structure could be a target of regulation.

The observation that the LVFF sequence is inserted into the head group region of the membrane parallels the findings of Sanders and coworkers^19^ who proposed that LVFF insertion may facilitate its interaction with cholesterol. Both structural and functional studies indicate that cholesterol interacts directly with the β-CTF. Structurally, a cholesterol-binding site was identified in the JM region of the β-CTF between the extracellular and TM domains^19^. Cholesterol and cholesterol-rich domains were linked to γ-secretase activity and the level of secreted Aβ^34^. In our previous studies of C55 reconstituted into POPS:POPC membrane bilayers^18^, we found that the largest changes upon cholesterol addition were at Phe19 and Gly25. The changes in membrane-embedded Phe19 would agree with a direct interaction between C99 and cholesterol. However, parallel FTIR studies found no large differences in the 1626 cm^−1^ β-sheet band in the presence or absence of cholesterol suggesting that cholesterol is not regulating the binding of the extracellular sequence to the membrane^18^. Nevertheless, the insertion of Tyr10 into the head group region of the bilayer may play a role in cholesterol binding. The common cholesterol recognition/interaction amino acid consensus (CRAC) motif described for cholesterol binding contains a tyrosine followed by a lysine or arginine within five residues^35^.

The structure of the extracellular region of C99 also has implications for the structure of Aβ monomers, oligomers and fibrils. γ-secretase cleavage of C99 releases monomers of the Aβ peptides into the extracellular environment where they associate into oligomers that rearrange to form protofibrils and mature fibrils. The LVFF region of both Aβ40 and Aβ42 in mature fibrils interacts with the hydrophobic C-terminus of the Aβ peptide after it is released from the membrane bilayer following γ-secretase cleavage. Additionally, the LVFF sequence forms parallel and in-register β-sheet via hydrogen bonding interactions with adjacent molecules along the fibril axis during fibril formation^36^. Importantly, the N-terminal region of the Aβ peptide including the YEV sequence is not ordered in fibrils or (in the case of a recent cryoEM structure of Aβ42^37^) bends around to allow the N-terminus to interact with C-terminus. This suggests that loss of the YEV – LVFF hairpin occurs during the conversion of membrane-bound Aβ into soluble protofibrils and fibrils. In fact, several studies have concluded that the N-terminus competes with the hydrophobic C-terminus of Aβ in forming a hydrophobic cluster, and consequently slows the formation of mature fibrils^38^. Our studies indicate that the β-sheet structure involving the N-terminus is induced by membrane binding and is retained in Aβ monomers and oligomers (see also^39^). Together, our current results and these previous studies argue that release of the hydrophobic Aβ C-terminus from the membrane bilayer upon cleavage of the membrane spanning domain of C99 sets into motion the rearrangement of interactions involving the critical LVFF motif, which ultimately leads to the release of N-terminal interactions as fibrils form.

## Methods

### Materials

^13^C-labeled amino acids were purchased from Cambridge Isotope Laboratories (Andover, MA). Other amino acids and octyl-β-glucoside were obtained from Sigma-Aldrich (St. Louis, MO). DMPC, DMPG, POPC and POPS were obtained from Avanti Polar Lipids (Alabaster, AL) as lyophilized powders and used without further purification. Human APP-specific antibody (WO-2) was obtained from Millipore (Billerica, MA). All cell culture reagents were from Invitrogen (Carlsbad, CA). The plasmids used to express human APP695 or β-CTF in eukaryotic cells have been previously described^40^. The different mutants were generated by site-directed mutagenesis according to manufacturer’s instructions (Quick-Change™, Stratagene, La Jolla, CA).

### Cell Cultures and Transfection

CHO cells were cultured in F12 medium supplemented with 10% fetal bovine serum and antibiotics at 37 °C and 5% CO_2_ and transfected 24 h after seeding as previously described^40^.

### Western Blotting

Western blotting was performed on cell lysates (10 µg protein). Membranes were incubated at 4 °C overnight with APP C-terminal (APP Cter) polyclonal antibody (1:5000), washed and incubated with 1:10,000 secondary anti-rabbit antibody conjugated to horseradish peroxidase, followed by ECL revelation (Amersham, Uppsala, Sweden).

### AICD Reporter Gene Assay

AICD release was measured by a reporter gene assay previously described^41^. CHO cells were transfected with C99, Tip60Gal4, Fe65 expression vectors, Gal4RE-luc and *Renilla* luciferase vectors. Luciferase activity was measured 48 h after transfection by the Dual Glo® luciferase assay (Promega, Madison, WI) Luciferase activity corrected for transfection efficiencies was calculated as the firefly/*Renilla* luciferase ratio.

### ECLIA Assays

Aβ production was monitored in the culture media 48 h after transfection. Briefly, samples were cleared by centrifugation (12,000 *g*, 3 min, 4 °C). Aβ38/Aβ40/Aβ42 were quantified in 25 µl of cellular medium by multiplex 4G8 or 6E10 Aβ ECLIA assays according to the manufacturer’s instructions (MesoScale Discovery, Gaithersburg, MD).

### Statistical Analyses

Statistical analyses were performed using GraphPad Prism™ software (GraphPad, La Jolla, CA). Statistical significance was evaluated by One-way ANOVA followed by Dunnett’s post-hoc test or as indicated.

### Peptide Synthesis and Purification

C55 peptides (Asp1-Lys55) corresponding to the TM and juxtamembrane regions of APP were synthesized by solid-phase methods (Keck Facility, Yale University). The purity was confirmed with MALDI-TOF mass spectrometry and analytical reverse phase HPLC.

### Protein Expression and Purification

The C55 sequence was subcloned into a pET21a vector and expressed with an added methionine encoded at the N-terminus and a linker plus His-tag (KLAAALEHHHHHH) at the C-terminus. This vector was used in the transformation of BL21 strain of *E. coli*. The transformed cells were plated on Luria Broth (LB) plates with ampicillin resistance and incubated at 37 °C overnight. A single colony from the plate was then selected and seeded into 20 ml of LB overnight. The resulting cell culture was used to inoculate 500 ml of LB with vigorous shaking at 37 °C. When the OD_600_ reached 0.8, 1 mM of isopropyl thiogalactoside was added to induce the overexpression of the proteins at 23 °C for 16–20 hours. The cells were harvested by centrifugation and broken using a French press. The C55 protein was purified with a Nickel NTA column using the following procedure. Cell pellets were washed 3 times with the 35 ml of lysis buffer (75 mM Tris, 300 mM NaCl, 0.2 mM EDTA, pH 7.8) until the washed solution became clear. The pellets were then dissolved in 35 ml of urea/SDS buffer (20 mM Tris, 150 mM NaCl, 8 M urea, 0.2% SDS, pH 7.8) and mixed overnight at room temperature. The resulting solution was centrifuged at 25,000 *g* for 20 min. The supernatants were collected and mixed with the pre-equilibrium Nickel beads for 2 hours at room temperature. The column was then washed with urea/SDS buffer and the proteins were eluted with 250 mM imidazole in Tris-buffered saline solution (pH 7.8) containing octyl-β-D-glucoside. The eluted fractions were collected and checked by SDS-PAGE. The protein purity was further confirmed with SDS-PAGE and mass spectroscopy.

### Reconstitution of C55 and C39 into Membrane Bilayers

Peptides were reconstituted into DMPC:DMPG or POPC:PS bilayers using methods described previously^18^. Briefly, the peptides were cosolubilized with lipids and octyl-β-glucoside in hexafluoroisopropanol. The organic solvent is removed under vacuum and the peptide-lipid-detergent mix is solubilized in buffer. The high critical micelle concentration of octyl-β-glucoside relative to other detergents makes it easy to slowly dialyze detergent away from the lipid and peptide, which greatly reduces peptide aggregation during the reconstitution process. The peptide:lipid molar ratio was 1:50 and the molar ratios between DMPC:DMPG or POPC:PS was 10:3, similar to the ratio of neutral-to-negatively charged lipids in plasma membrane. The mixture was then incubated for 3 h, frozen and put under vacuum overnight to remove the solvent. The dried peptide-lipid-detergent mixture was rehydrated by dissolving in HEPES buffer (5 mM HEPES, 50 mM NaCl, pH 7.2) and the detergent was removed by dialysis.

### ATR-FTIR Spectroscopy

Polarized attenuated total reflection (ATR) FTIR spectra were obtained on a Bruker IFS 66 V/S spectrometer. ATR-FTIR spectroscopy was used to characterize the global secondary structure C55 and C39 in bilayers. The wild-type and mutant peptides reconstituted in bilayers were layered down on a germanium plate and bulk water was removed by a gentle strain of nitrogen gas.

### Fluorescence Spectroscopy

Tryptophan fluorescence measurements on C55 F19W and F20W with and without LUVs were performed using a Horiba Jobin Yvon Fluorolog FL3-22 spectrofluorimeter. The samples were placed into a quartz cuvette with a path length of 5 mm and fluorescence was immediately measured. Spectra were collected from 300–450 nm.

### Solid-State NMR Spectroscopy

2D dipolar assisted rotational resonance (DARR) NMR experiments were performed at a ^13^C frequency of 125 MHz on a Bruker AVANCE spectrometer. The MAS spinning rate was set to 9–11 KHz ( ± 5 Hz) depending on whether the spinning sidebands will overlap with the labeled ^13^C frequencies. The ramped amplitude cross polarization contact time was 2 ms. Two-pulse phase-modulated decoupling was used during the evolution and acquisition periods with a radiofrequency field strength of 80 kHz. The DARR mixing time was 600 ms. The sample temperature was maintained at 198 K (±2 K).

REDOR NMR experiments^20^ were performed at a ^13^C frequency of 242 MHz on a Bruker AVANCE spectrometer with a dephasing period of 20 rotor cycles at 10 KHz MAS rate (2 ms). The REDOR filtered spectra (ΔS) were obtained by subtracting spectra with (S) and without (S_0_) rotor-synchronized ^15^N π pulses (10–11 μs). To reduce artifacts, S and S_0_ spectral acquisition was interleaved and difference spectra were acquired scan-by-scan. ΔS spectra were summed over 60–100 K scans using ~5–6 mgs of rhodopsin in a typical sample.

### Data Availability

The datasets generated during and/or analyzed during the current study are available from the corresponding author on reasonable request.

## Electronic supplementary material

Supporting Information
