# Supplementary material for: β-Sheet Structure within the Extracellular Domain of C99 Regulates Amyloidogenic Processing
Source: Sci Rep. 2017 Dec 7;7:17159. doi: 10.1038/s41598-017-17144-0 (PMC5719365; doi:10.1038/s41598-017-17144-0)
Supplement: Supplementary file 1 — Supporting Information [file 41598_2017_17144_MOESM1_ESM.pdf]

SUPPORTING INFORMATION

β-Sheet Structure within the Extracellular Domain of C99 Regulates Amyloidogenic Processing

Yi Hu[1], Pascal Kienlen-Campard[2], Tzu-Chun Tang[1], Florian Perrin[2,3], Rémi Opsomer[2], Marie Decock[2], Xiaoshu Pan[1], Jean-Noel Octave[2], Stefan N. Constantinescu[3,*] and Steven O. Smith[1,*]

[1]Department of Biochemistry and Cell Biology, Stony Brook University, Stony Brook, NY 11794. [2]Institute of Neuroscience, Université catholique de Louvain, Brussels 1200, Belgium. [3]Ludwig Institute for Cancer Research and de Duve Institute, Université catholique de Louvain, Brussels 1200, Belgium

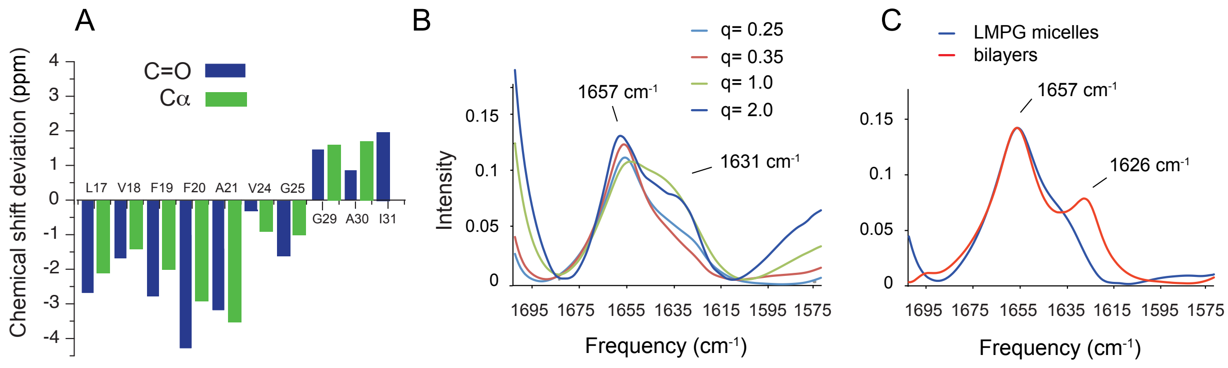

Figure S1. Extracellular region of C55 forms β-sheet in membrane bilayers. (A) ^{13}C Chemical shifts of selected Cα and C=O resonances between Leu17 and Ile31 from solid-state NMR measurements [1]. The chemical shift deviation is plotted between the observed chemical shift and the average chemical shifts from the Biological Magnetic Resonance Bank data base. Deviations to lower chemical shift values correspond to β-sheet secondary structure. Deviations to higher chemical shift values correspond to α-helical secondary structure. For wild-type C55, the Cα and C=O resonances between Leu17 and Ala21 all exhibit negative deviations corresponding to β-strand or sheet structure. The observation of β-structure is consistent with the FTIR results presented in Figure 3, but contrasts with solution NMR structures previously determined of this region [2,3].

The bilayer environment induces β-structure in C55 [1]. (B) Comparison of FTIR spectra of wild-type C55 reconstituted into bicelles composed of DMPC:DMPG:DHPC with q values ranging from q = 0.25, 0.35, 1.0, and 2.0. The q value is the ratio of long acyl chain lipids (DMPC and DMPG) to short acyl chain lipids (DHPC) [4,5]. Bicelles with small q values (e.g. 0.25) behave like

detergents, while bicelles with large q values (e.g. 2.0) behave like bilayer membranes. (C) Comparison of FTIR spectra of wild-type C55 reconstituted in membrane bilayers with the spectra of C55 solubilized in LMPG detergent. In (B and C), the membrane bilayer spectra were obtained using a molar ratio of the DMPC:DMPG of 10:3, and the molar ratio of protein: total lipid was 1:50. The difference in behavior of hydrophobic peptides in detergent and membrane environments is related to the large differences between micelles and bilayers, which start with the differences in their overall geometry and curvature. These structural differences influence the dynamics and lateral pressure of the hydrophobic acyl chains as well as the accessibility of water within the hydrophobic core of bilayers. All of these factors influence membrane protein structure.

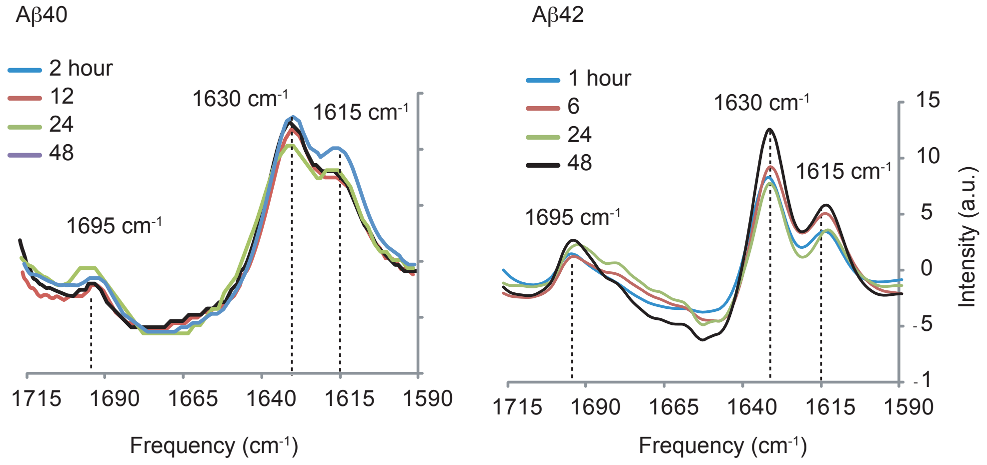

Figure S2. Aβ40 and Aβ42 exhibit stable anti-parallel structure upon binding to membrane vesicles. FTIR difference spectra obtained as a function of time are shown of Aβ40 and Aβ42 added to large unilamellar vesicles (100 nm diameter) composed of dimyristoylphosphocholine (DMPC) and dimyristoylphosphoglycine (DMPG) in a 10:3 molar ratio. Each difference spectrum was obtained by subtracting the t = 0 spectrum. The spectra shown represent a rapid conversion from random coil structure (characterized by the broad band between 1650-1680 cm^{-1}) to anti-parallel β-structure within 1-2 hours in the presence of lipid. The difference spectra highlight the ~1615 cm^{-1} and ~1695 cm^{-1} bands characteristic of anti-parallel secondary structure. The 1695 cm^{-1} band is often obscured by the random coil band in Aβ spectra (or the helical 1655 cm^{-1} band in C55 spectra), but is well pronounced in difference spectra. The 1615 cm^{-1} band results from the incorporation of a ^{13}C-labeled carbonyl at Gly33. The incorporation of a $^{13}C{=}O$ label results in a lower frequency amide I band, which has enhanced intensity when the backbone is in antiparallel structure. A similar use of ^{13}C backbone labeling was previously used to characterize anti-parallel structure in C55 [1] and in Aβ42 [6]. These bands are stable over 48 h at 37 °C. In contrast, the anti-parallel bands only transiently appear (2-6 h) in Aβ40 and Aβ42 in solution (i.e. in the absence of membrane vesicles) as these peptides convert to fibrils that have a parallel, in-register orientation of the β-strands within the fibrils [6].

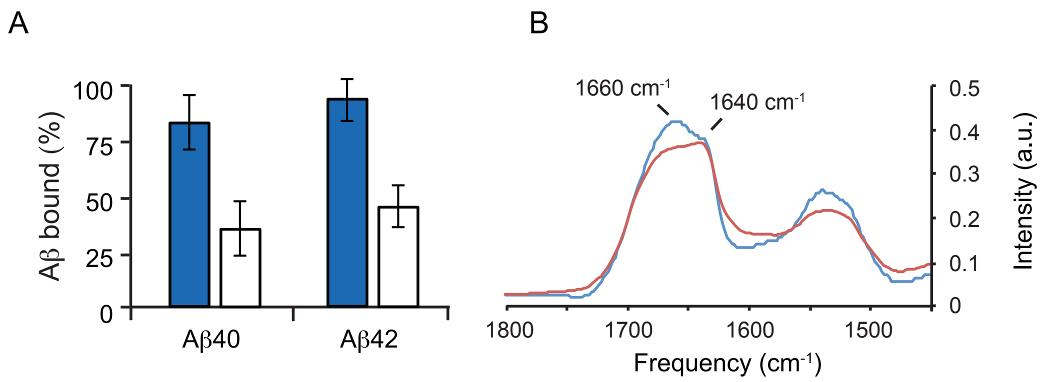

Figure S3. Mutation of the LVFF sequence to AAAA in Aβ40 and Aβ42 reduces binding to DMPC:DMPG (10:3 molar ratio) membrane vesicles. (A) Membrane binding of Aβ40-WT and Aβ42-WT (dark blue) and Aβ40-AAAA and Aβ42-AAAA (white). Peptide binding was carried out using a centrifugation assay making use of sucrose-loaded vesicles [7,8]. Aβ40 and Aβ42 monomers (25 mM) were added to 100 nm lipid vesicles (10 mM) at room temperature. Vesicle-bound peptide was removed from solution by centrifugation. The amount of Aβ peptide remaining in solution was assayed by lyophilizing the supernatant and quantifying the peptide by HPLC. (B) FTIR spectra of soluble Aβ40-AAAA (blue) and Aβ42-AAAA (red) obtained after 48 h of incubation at 37 °C. The broad band at ~1660 cm^{-1} and small feature at 1640 cm^{-1} are characteristic of largely random coil secondary structure indicating that these peptides do not form amyloid fibrils.

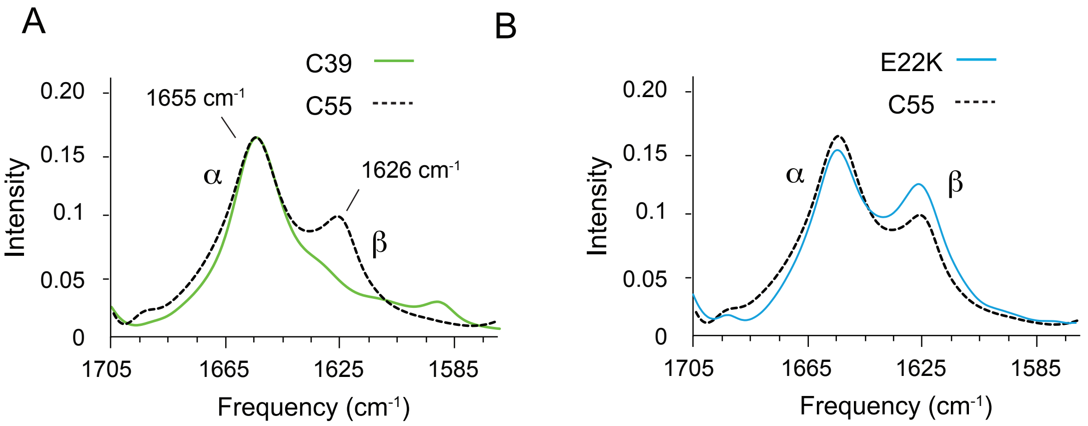

Figure S4. Comparison of FTIR spectrum of wild-type C55 with wild-type C39 (A) and E22K C55 (B). C39 corresponds to the extracellular and TM domains of the α-CTF. Comparison of C39 (green line) with C55 (dashed line) shows that the β-sheet band at 1626 cm^{-1} is lost upon deletion of the N-terminal 16 amino acids of C55 arguing that the LVFFAE sequence alone (i.e. in the context of the α-CTF) is not able to form anti-parallel structure. Comparison of wild-type C55 (dashed line) with the E22K mutant of C55 (blue line) suggests that the anti-parallel structure in the extracellular region of C55 does not arise from pairing of the KLVFFAE sequence on different peptides as observed in short peptides corresponding to only this sequence [9]. Deconvolution of the amide I region of the E22K spectrum suggests that 9-12 residues of the extracellular sequence contribute to the β-sheet, consistent with the three-stranded β-sheet structure in the main text.

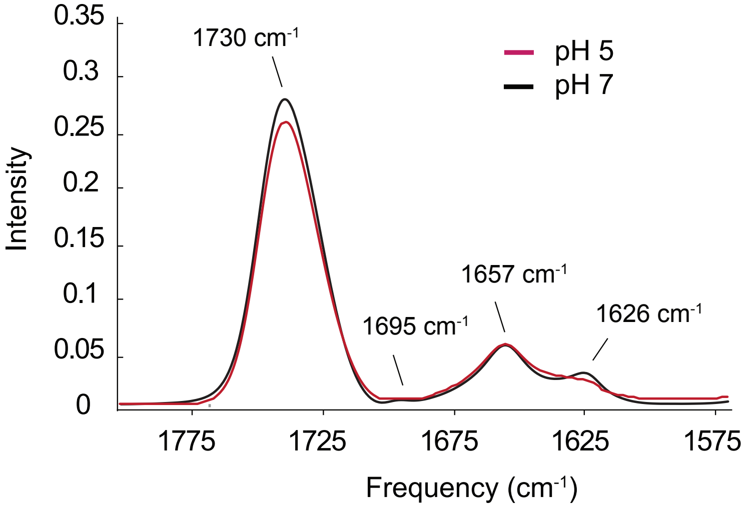

Figure S5. Influence of pH on the β-sheet structure of C55. FTIR spectra were obtained of C55 reconstituted into membrane bilayers composed of POPC:PS (10:3 molar ratio) at pH 5 and pH 7. The amide I resonance at 1626 cm^{-1} assigned to β-sheet secondary structure appears to broaden and lose intensity at lower pH. The proposed β-sheet structure involves complementary charge pairing at pH 7 that may be disrupted at lower pH.

A
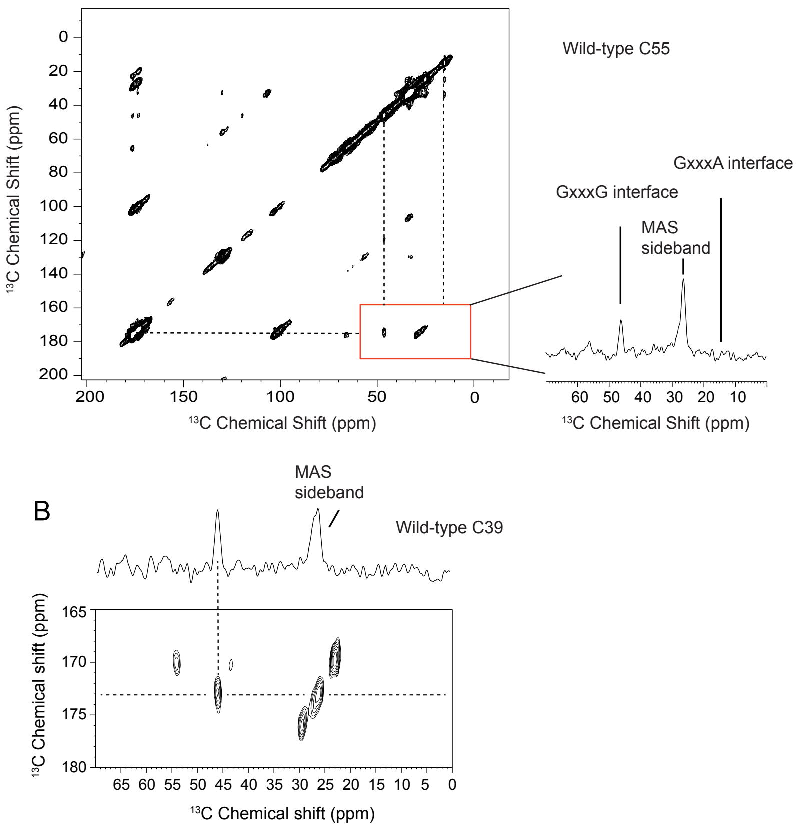

Figure S6. Magic angle spinning NMR of C55 and C39. Dimer contacts probed using 2D DARR NMR. (A) DARR NMR spectra were obtained of the C55 peptide reconstituted into POPC:PS bilayers. These experiments involve co-mixing of two different peptides where cross peaks in the 2D spectra are only observed between heterodimers. Specifically, ^{13}C labels were selectively incorporated at Gly33 and Ala42 in two different C55 peptides (peptide 1 contains Gly33 ^{13}C=O and Ala42 ^{13}Cβ and peptide 2 contains Gly33 ^{13}Cα and Ala42 ^{13}C=O). In the heterodimer, the Gly33 residues are closely packed if the $G^{33}xxxG^{37}$ motif mediates dimerization, whereas the Ala42 residues are in close proximity if the $G^{38}xxxA^{42}$ motif mediates dimerization. Close proximity of ^{13}C labels (< 6 Å) is manifested as a cross peak in the 2D NMR spectrum. Panel (A) shows the full 2D spectrum, where the region containing cross peaks is boxed in red. For the wild-type TM peptide, the cross peak between the ^{13}Cα carbon of Gly33 at 45 ppm and the ^{13}C=O carbon at 175 ppm is observed (red box). A row through this region of the spectrum clearly reveals the cross peak (inset). There is no cross peak between the Ala42 positions. (B) DARR NMR spectra were obtained of the C39 peptide reconstituted into POPC:PS bilayers using the same ^{13}C-labeling scheme as in (A). The increase in the intensity of the Gly33-Gly33 cross peak relative to the MAS sideband indicates that C39 forms a stronger dimer than C55.

References

1 Tang, T. C. *et al.* Conformational changes induced by the A21G Flemish mutation in the amyloid precursor protein lead to increased Aβ production. *Structure* **22**, 387-396, doi:10.1016/j.str.2013.12.012 (2014).

2 Nadezhdin, K. D., Bocharova, O. V., Bocharov, E. V. & Arseniev, A. S. Dimeric structure of transmembrane domain of amyloid precursor protein in micellar environment. *FEBS Lett.* **586**, 1687-1692, doi:10.1016/j.febslet.2012.04.062 (2012).

3 Beel, A. J. *et al.* Structural studies of the transmembrane C-terminal domain of the amyloid precursor protein (APP): Does APP function as a cholesterol sensor? *Biochemistry* **47**, 9428-9446 (2008).

4 Sanders, C. R. & Landis, G. C. Reconstitution of membrane proteins into lipid-rich bilayered mixed micelles for NMR studies. *Biochemistry* **34**, 4030-4040, doi:doi:10.1021/bi00012a022 (1995).

5 Kim, H. J., Howell, S. C., Van Horn, W. D., Jeon, Y. H. & Sanders, C. R. Recent advances in the application of solution NMR spectroscopy to multi-span integral membrane proteins. *Progress in Nuclear Magnetic Resonance Spectroscopy* **55**, 335-360 (2009).

6 Fu, Z., Aucoin, D., Davis, J., Van Nostrand, W. E. & Smith, S. O. Mechanism of nucleated conformational conversion of Ab42. *Biochemistry*, doi:10.1021/acs.biochem.5b00467 (2015).

7 Buser, C. A., Sigal, C. T., Resh, M. D. & McLaughlin, S. Membrane binding of myristylated peptides corresponding to the NH2 terminus of Src. *Biochemistry* **33**, 13093-13101, doi:doi:10.1021/bi00248a019 (1994).

8 Ben-Tal, N., Honig, B., Peitzsch, R. M., Denisov, G. & McLaughlin, S. Binding of small basic peptides to membranes containing acidic lipids: Theoretical models and experimental results. *Biophys. J.* **71**, 561-575 (1996).

9 Balbach, J. J. *et al.* Amyloid fibril formation by $A\beta_{16-22}$, a seven-residue fragment of the Alzheimer's β-amyloid peptide, and structural characterization by solid state NMR. *Biochemistry* **39**, 13748-13759, doi:doi:10.1021/bi0011330 (2000).
